# Supplementary material for: Partial asynchrony of coniferous forest carbon sources and sinks at the intra-annual time scale
Source: Nat Commun. 2024 Aug 5;15:6169. doi: 10.1038/s41467-024-49494-5 (PMC11300610; doi:10.1038/s41467-024-49494-5)
Supplement: Supplementary file 1 — Supplementary Information [file 41467_2024_49494_MOESM1_ESM.pdf]

Supplementary Information

PARTIAL ASYNCHRONY OF CONIFEROUS FOREST CARBON SOURCES AND SINKS AT THE INTRA-ANNUAL TIME SCALE.

Supplementary methods

Site selection and dataset assembly

Wood formation

This study uses wood formation data collected in 81 sites belonging to boreal, temperate and Mediterranean biomes. All the data followed the criteria or procedures applied either in the field or laboratory, as described below and according to the methodology described by Rossi, Anfodillo, et al. (2006); Rossi, Deslauriers, et al. (2006).

The timings of wood formation were determined by monitoring healthy dominant trees. The sample size ranged from 1 to 55 trees among all sites throughout the entire growing seasons of 1998 to 2018. Stem microcores were collected weekly, or occasionally biweekly, at breast height (i.e., 1.3 m) using surgical bone-sampling needles or a Trephor tool. The samples included mature and developing xylem of the current year, the cambial zone and adjacent phloem, and at least one previous complete tree ring. The microcores were fixed in propionic or acetic acid solutions mixed with formaldehyde and stored in ethanol-water at 5 °C. They were then dehydrated with successive immersions in ethanol and D-limonene and finally embedded in paraffin or glycol methacrylate (samples from Switzerland were not embedded). The microcores were cut with rotary or sledge microtomes to obtain 10 to 30 μm thickness transverse sections.

The sections obtained were stained with cresyl violet acetate or a mixture of safranin and astra/Alcian blue, then examined by light microscopy (bright-field and polarised light). Cambial initials of the vascular cambium, a secondary meristem, divide outward and inward to produce phloem and xylem mother cells that, in turn, form new phloem and xylem tissues. The process of tracheid formation (i.e., cell differentiation) is divided into different phenological phases including cell enlargement, secondary cell wall thickening and lignification, and then programmed cell death, leading to the mature stage. Cambial

cells are characterised by thin cell walls and small radial diameters. The enlargement zone is represented by the absence of glistening under polarised light, which indicates the presence of only primary cell walls. Cells undergoing secondary cell wall formation glistened under polarised light. In mature cells, Cresyl violet acetate reacts with lignin, turning from violet to blue. Maturation is reached when the cell walls are entirely blue.

To investigate the variations in climate among the locations where we monitored wood formation, we sourced bioclimatic data from the CHELSA bioclimatic database V2.1, offering a spatial resolution of 30 arcseconds. From the set of 19 bioclimatic parameters, we curated a selection of seven variables, as detailed in the Supplementary Table 1. We excluded parameters that delivered overly broad climate descriptions, like annual temperature and precipitation, and filtered out variables that exhibited strong autocorrelations (with a correlation coefficient exceeding |0.7|). To categorize our study sites based on their climate-related attributes, we applied the Partitioning Around Medoids clustering algorithm (PAM), an extension of k-means clustering approach. We employed the Within-Sum-of-Squares method (WSS) to identify the optimal number of clusters. This method minimizes the internal distances between data points within each cluster. We determined that four clusters best suited the task of grouping our wood formation study sites effectively (Supplementary Figure 1). We proceeded to perform a Principal Component Analysis (PCA) on the selected bioclimatic variables. This analytical technique allowed us to visualize and understand the climatic positioning of our 81 wood formation sites in relation to these variables. Pearson's correlation coefficient was employed to identify the climatic factors that influenced the ordering of wood formation study sites by principal components (Supplementary Table 1).

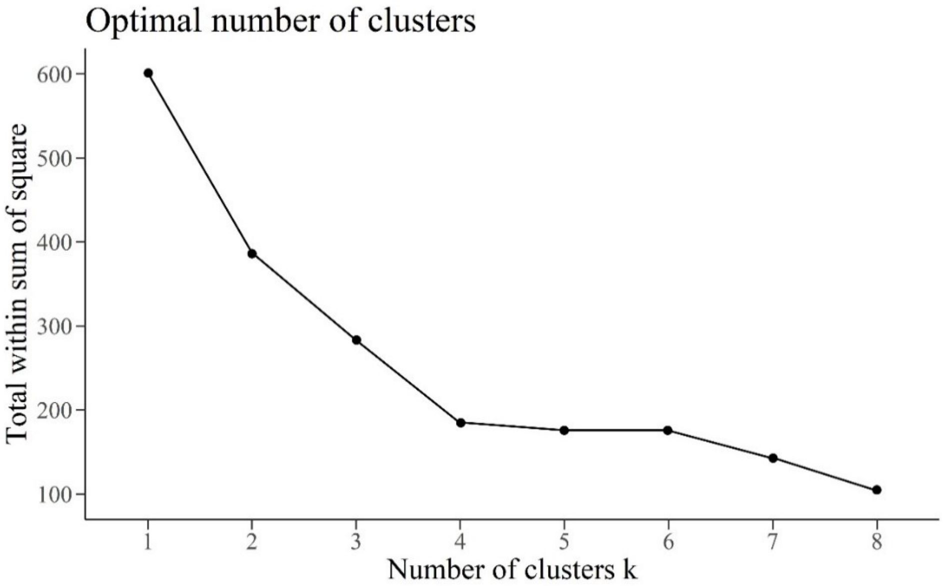

Supplementary Figure 1 Within Sum of Square (WSS) of the optimal number of clusters determined for climate-related wood formation sites grouping.

Supplementary Table 1 Pearson correlation coefficients between climatic variable and the first two principal components, eigenvalues, and variance explained.

Bioclimatic variables	Abbreviation	PC1	PC2
Temperature seasonality (STD x 100)	bio4	-0.43	-0.82
Mean temperature of the driest quarter	bio9	0.88	0.38
Mean temperature of the warmest quarter	bio10	0.84	-0.14
Mean temperature of the coldest quarter	bio11	0.82	0.54
Precipitation seasonality (CV)	bio16	-0.55	0.63
Precipitation of the driest quarter	bio17	-0.56	0.50
Precipitation of the warmest quarter	bio18	-0.72	0.53
Eigenvalue		3.49	2.08
Variance explained		49.90	29.73

In the main text of this work and for all the following analysis, data referring to the phenological phases of cambial activity, cell enlargement, and cell wall thickening and

lignification were grouped by biome and site and normalized (min-max normalisation) on a scale from 0 to 1.

NSC

We assembled the NSC dataset by sub-setting the conifers section of the dataset used by Martínez-Vilalta et al. (2016) and updating it with more recent data, finally resulting in 57 sites distributed in boreal, temperate and Mediterranean biomes. The studies included seasonal NSC data on wild species measured under natural field conditions. When studies involved experimental manipulations, we only considered results from unmanipulated controls. In addition, to ensure good temporal coverage and reduce unwanted variability due to some specific characteristics of the samples, we selected only work that fulfilled the following criteria: (1) study duration was at least four months, (2) the same individuals or populations were measured at least three times spanning the length of the study, (3) plants were mature, (4) measurements were taken on needles, main stem, fine or coarse roots (5) values reported were starch/fructans, or soluble sugars. All individual NSC data points were extracted from the text, tables, or figures of each study or by contacting authors directly, in the latter case using the software GetData Graph Digitizer (Version 2.26). Usually, NSC concentrations are expressed as % or mg/g dry mass directly, otherwise, values were converted to mg/g. Finally, NSC concentration was grouped by biome, organ, study, extraction and quantification method. These two latter grouping operations were performed given that a study on the comparability of NSC measurements across laboratories concludes that NSC estimates for woody plant tissues may not be directly comparable (Quentin et al., 2015). Ultimately, all data were normalized (min-max normalisation) on a scale from 0 to 1.

Flux data evergreen needleleaf forests (FLUXNET 2015)

We used tier-one level data from the FLUXNET2015 dataset (Pastorello et al., 2020) and extracted data at daily temporal aggregation from ENF (Evergreen Needleleaf Forests) sites. These sites consist of forest lands dominated by woody vegetation with a cover of

>60% and height exceeding 2 meters. In addition, to reduce unwanted variability due to some specific characteristics of the site, we selected only data in which stands (1) belonged to boreal, temperate, or Mediterranean biomes, (2) were at least 15 years old, and (3) were not recently disturbed (e.g., burn sites). The dataset finally consisted of 39 sites. CO_2 fluxes extracted for each site were Net Ecosystem Exchange (NEE), Ecosystem Respiration (RECO), and Gross Primary Production (GPP). Specifically, for the NEE we used the variable NEE_VUT_REF since it maintains the temporal variability and represents the ensemble (Pastorello et al., 2020). There are two main ways to estimate GPP and RECO (in units of g C m^{-2} d^{-1}) by partitioning of the NEE computed with the variable Ustar (u^*) threshold (VUT): (1) night-time method (GPP_NT_VUT_REF and RECO_NT_VUT_REF); and (2) day-time method (GPP_DT_VUT_REF) (Pastorello et al., 2020). All results shown here use an average of the night- and day-time partitioning methods (RECO_NT_VUT_REF and RECO_DT_VUT_REF for RECO, GPP_NT_VUT_REF and GPP_DT_VUT_REF for GPP). Finally, C fluxes were grouped by biome, flux type (i.e., NEE, GPP, RECO), and site and normalized (min-max normalisation) on a scale from 0 to 1.

Flux data in wood formation sites (FLUXSAT V2.0)

Daily GPP data were extracted for each site and each year where the wood formation was monitored. Differently from the dataset obtained with the data from FLUXNET2015, in this case, while the samples were collected on coniferous species, the study area could consist of a mixed forest. These GPP products were extracted by FluxSat v2.0 (Joiner & Yoshida, 2021), where FluxSat refers to data derived using FLUXNET eddy covariance tower site data and the coincident satellite data. This dataset provides global gridded daily estimates of terrestrial GPP and uncertainties at 0.05° resolution for the period 01/03/2000 to the recent past (Joiner & Yoshida, 2021). The GPP was derived from the MODerate-resolution Imaging Spectroradiometer (MODIS) instruments on the NASA Terra and Aqua satellites using the Nadir Bidirectional Reflectance Distribution Function (BRDF)-Adjusted Reflectances (NBAR) product as input to neural networks that were used to globally upscale GPP estimated from selected FLUXNET 2015 eddy covariance

tower sites. GPP value (in g C m^{-2} d^{-1}) for each site resulted from the interpolation of GPP values of the four nearest pixels (Joiner & Yoshida, 2021).

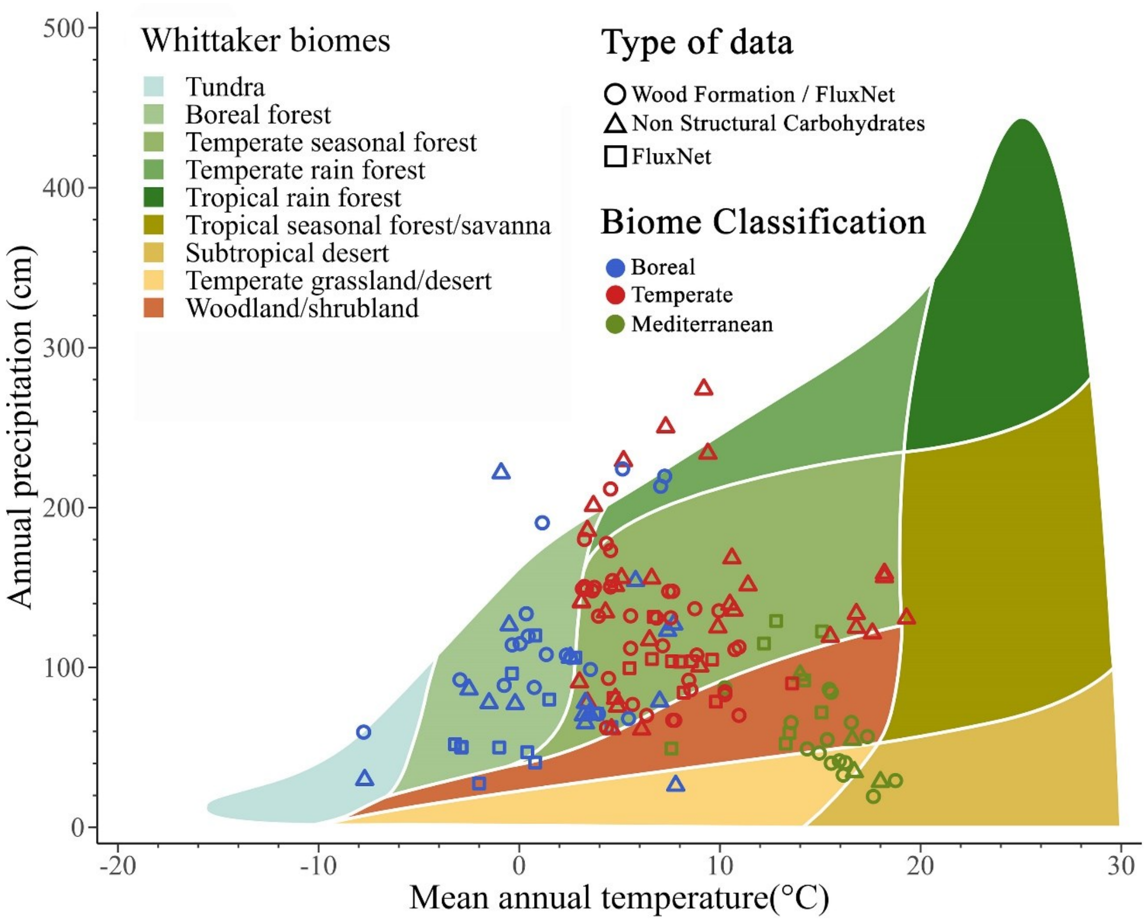

Supplementary Figure 2 Whittaker biome plot showing the comparison between the study site classification in the present study (i.e. boreal, temperate and Mediterranean biomes) and Whittaker biome classification according to mean annual temperature (°C) and annual precipitation (cm).

Supplementary Table 2 Alphabetical list of all the species monitored in the study. In total, 39 species of conifers belonging to 8 genera were the object of the study.

Wood formation species	Non-structural carbohydrates species
Abies alba Mill.	*Abies alba* Mill.
Abies balsamea (L.) Mill.	*Abies lasiocarpa* (Hook.) Nutt.
Abies georgei (var. *smithii*)	*Juniperus occidentalis* Hook.
Abies pindrow (Royle ex D.Don) Royle	*Larix decidua* Mill.
Cedrus libani A. Rich	*Picea abies* L. Karst
Juniperus przewalskii Kom.	*Picea engelmanii* Parry ex Engelm.
Juniperus thurifera L.	*Picea glauca* (Moench) Voss
Larix decidua Mill.	*Picea rubens* Sarg.
Picea abies L. Karst	*Picea sitchensis* (Bongard) Carrière
Picea mariana (Mill.) B.S.P.	*Pinus banksiana* Lamb.
Pinus cembra L.	*Pinus cembra* L.
Pinus flexilis James.	*Pinus contorta* Douglas ex Loudon
Pinus halepensis Mill.	*Pinus elliottii* Engelm.
Pinus heldreichii Christ	*Pinus halepensis* Mill.
Pinus longaeva Bailey	*Pinus palustris* Mill.
Pinus massoniana Lamb.	*Pinus pinaster* Ait.
Pinus peuce Griseb.	*Pinus ponderosa* Douglas ex Lawson
Pinus pinaster Ait.	*Pinus radiata* D.Don
Pinus ponderosa Douglas ex Lawson	*Pinus sylvestris* L.
Pinus strobus L.	*Pinus taeda* L.
Pinus sylvestris L.	*Pseudotsuga menziesii* (Mirb.) Franco
Pinus tabulaeformis Carr.	*Tsuga canadensis* (L.) Carrière
Pinus uncinata Mill. Ex Mirb	*Tsuga heterophylla* (Raf.) Sarg.
	Tsuga mertensiana (Bong.) Carr.

Supplementary Note 1: Bioclimatic Analysis

The PCA of the 81 sites in which wood formation assessments were carried out yielded eight components, of which two were significant, collectively accounting for 79.63% of the variance (Fig. S3 and Supplementary Table 1). The first component (PC1), which explained 49.90% of the variance (Supplementary Table 1), showed a strong correlation ($r > |0.7|$) with the mean temperatures of the driest, warmest, and coldest quarters (bio9, bio10, and bio11, respectively), as well as the precipitation of the warmest quarter (bio18) (Supplementary Table 1). The second component (PC2), responsible for explaining 29.73% of the variance (Supplementary Table 1), displayed a strong correlation ($r > |0.7|$) with temperature seasonality (bio4). It also showed moderate correlations ($|0.5| > r > |0.7|$) with the mean temperature of the coldest quarter (bio11), precipitation seasonality (bio16), and precipitation of the driest and warmest quarters (bio17 and bio18, respectively) (Supplementary Table 1).

Partitioning Around Medoids clustering algorithm facilitated the distinction of four bioclimatic clusters within the Northern Hemisphere, with a clear border between climatic biomes (Fig. S3). The Mediterranean cluster showed direct correlations with the mean temperature of the driest, warmest, and coldest quarters (bio9, bio10, and bio11, respectively) while inversely correlating with precipitation seasonality (bio16) and precipitation of the driest and warmest quarters (bio17 and bio18, respectively) (Fig. S3). The sites located in temperate biomes belonged to two main clusters, with the colder sites being directly correlated with precipitation seasonality (bio16) and precipitation of the driest and warmest quarters (bio17 and bio18, respectively), and inversely correlated with the mean temperature of the driest, warmest, and coldest quarters (bio9, bio10, bio11, respectively) (Fig. S3). The central temperate cluster was associated with most bioclimatic variables used in the analysis. Lastly, the boreal cluster was primarily determined by temperature seasonality (bio4) (Fig. S3).

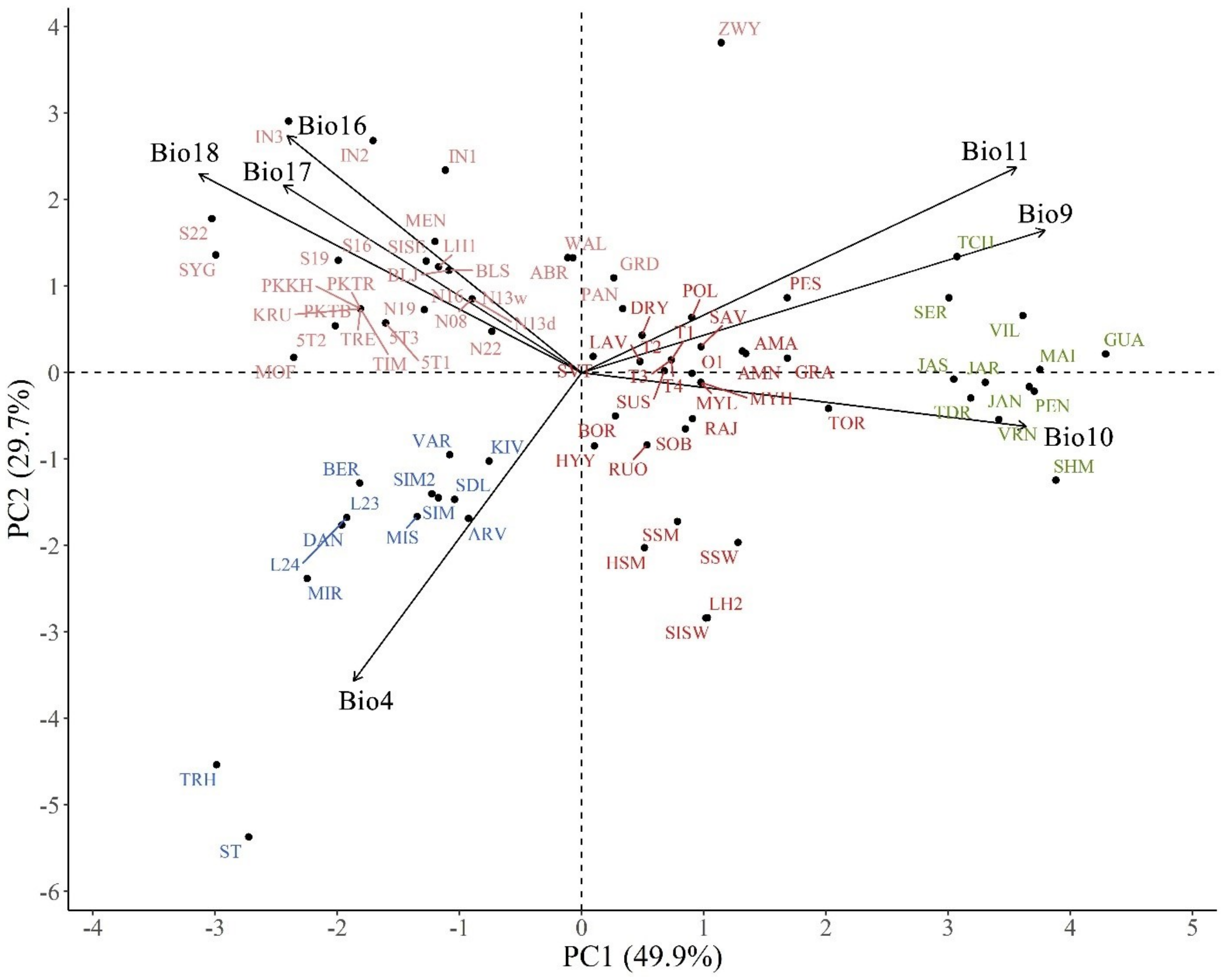

Supplementary Figure 3 Climate-related variability among 81 wood formation sites of 7 CHELSA (database V2.1) bioclimatic variables summarized in a PCA biplot. The color of the site ID is consistent with color codes used for bioclimatic clusters (boreal –blue labels–, temperate –red labels– and Mediterranean –green labels– biomes). Scores correspond to the first (PC1) and second (PC2) axes of the PCA.

Supplementary Note 2: Non-linear fittings

The seasonal dynamics of wood formation (i.e., cambial activity, cell enlargement, and cell wall thickening and lignification), NSC (i.e., starch and soluble sugars in needles, stem and roots), and carbon fluxes (i.e., NEE, GPP and RECO) were fitted with skewed normal distribution or V-type exponential curves with a residual standard error (RSE) ranging between 0.09 and 0.27 (Supplementary Table 3). In each fitting in the boreal biome, the peak is reached later than in other biomes, and the period of maximum activity (or minimum when considering soluble sugar concentrations) was shorter than in temperate and Mediterranean biomes that followed this specific order. Therefore, the amplitude of the oscillations among biomes for each fitting was larger in Mediterranean biomes than temperate and boreal biomes.

For each curve we estimated the timing of the maximum value (or minimum when considering soluble sugar concentrations) (Tables S4, S5, and S6). In addition to this, fitted curves pertaining of seasonal pattern of wood formation (i.e., cambial activity, cell enlargement, and cell wall thickening and lignification) and carbon fluxes derived from FluxNet data (i.e., NEE, GPP, and RECO) were utilized to estimate the timing of key percentiles, namely the 10th, 25th, 50th, 75th and 90th percentiles, for both the ascending and descending portions of the curves. We opted to use the 75th percentile as the threshold for defining the period of maximum activity (MA). However, when the curve exhibited a minimum in soluble sugar concentrations, the period of maximum activity was determined based on the 25th percentile.

For each function, the area under the curve (AUC) was quantified, integrating the function itself (Tables S4, S5, and S6). The process of integration was also repeated by calculating the AUC of the period of MA for each function. This latter was obtained by using a definite integral with an interval consisting of a lower and upper limit represented by the points of intersection used to identify 75% of the maximum value of the curve and defining the period of MA (Tables S4, S5, and S6). Supplementary Figure 7 presents a comparison among the AUC of the period of MA for the processes of cell enlargement, GPP, and cell wall thickening and lignification.

Supplementary Table 3 Summary of all curve fitting. RSE indicates the residual standard error. All fittings were significant, at least with a p-value < 0.05.

V type exponential					
Curve fitting			Parameters estimate (± Standard error)		
**Biome**	**Curve**	**RSE**	**Ymax**	**μ**	**Xmax**
Temperate	Needles soluble sugars	0.23	0.40(0.01)	1.02(0.01)	7.61(0.31)
Boreal	Needles soluble sugars	0.25	0.41(0.02)	1.03(0.01)	6.67(0.19)
Mediterranean	Needles starch	0.10	0.53(0.07)	0.42(0.06)	6.09(0.08)
Mediterranean	Needles soluble sugars	0.14	0.39(0.03)	1.01(0.01)	5.52(2.55)
Temperate	Stem soluble sugars	0.27	0.34(0.02)	1.01(0.01)	6.46(0.68)
Boreal	Stem starch	0.22	0.34(0.02)	0.95(0.02)	5.89(0.47)
Boreal	Stem soluble sugars	0.21	0.21(0.02)	1.02(0.01)	6.91(0.54)
Mediterranean	Stem starch	0.14	0.30(0.05)	0.97(0.02)	4.61(1.24)
Mediterranean	Stem soluble sugars	0.24	0.27(0.06)	1.02(0.01)	6.25(1.00)
Temperate	Roots soluble sugars	0.19	0.33(0.02)	1.01(0.01)	6.39(0.52)
Boreal	Roots starch	0.13	0.29(0.02)	0.95(0.01)	6.97(0.43)
Boreal	Roots soluble sugars	0.16	0.28(0.03)	1.03(0.01)	7.03(0.55)

Skewed normal					
Curve fitting			Parameters estimate (± Standard error)		
**Biome**	**Curve**	**RSE**	**ξ**	**ω**	**α**
Temperate	Needles starch	0.23	4.04(0.33)	2.62(0.31)	1.25(0.53)
Boreal	Needles starch	0.22	4.61(0.25)	-2.20(0.30)	-1.99(1.00)
Temperate	Stem starch	0.22	2.88(0.41)	4.47(0.68)	2.41(1.26)
Temperate	Roots starch	0.22	1.36(0.80)	6.15(1.38)	2.13(1.84)
Mediterranean	Roots starch	0.14	5.86(0.66)	3.79(1.47)	-2.44(2.01)
Temperate	Roots soluble sugars	0.25	11.76(0.57)	-5.09(1.08)	3.98(4.42)
Boreal	Cambial activity	0.18	143.78(0.72)	50.88(1.25)	2.91(0.26)
Boreal	Cell enlargement	0.20	155.63(0.54)	39.34(0.91)	2.88(0.21)
Boreal	Cell wall thickening and lignification	0.20	180.22(1.11)	43.70(1.34)	1.76(0.16)
Temperate	Cambial activity	0.21	117.39(1.02)	72.14(1.69)	2.85(0.25)
Temperate	Cell enlargement	0.20	158.48(2.72)	37.57(3.98)	0.60(0.41)
Temperate	Cell wall thickening and lignification	0.21	180.99(2.61)	54.83(2.55)	1.31(0.19)
Mediterranean	Cambial activity	0.25	27.94(19.01)	171.02(15.27)	2.63(1.99)
Mediterranean	Cell enlargement	0.23	163.83(2.39)	51.94(2.55)	0.01(2.6)
Mediterranean	Cell wall thickening and lignification	0.26	155.88(3.46)	83.25(3.98)	2.03(055)
Temperate	GPP	0.13	229.69(2.85)	98.90(1.44)	-0.67(0.05)
Boreal	GPP	0.11	228.37(1.20)	66.66(0.72)	-0.81(0.04)
Mediterranean	GPP	0.12	80.91(2.96)	161.98(3.18)	1.62(0.09)
Temperate	RECO	0.12	261.51(1.01)	106.65(0.90)	-1.26(0.04)
Boreal	RECO	0.09	244.30(0.72)	68.56(0.56)	-1.09(0.03)
Mediterranean	RECO	0.12	75.57(3.25)	191.75(5.82)	2.08(0.17)
Temperate	NEE	0.20	99.74(0.53)	103.16(0.88)	2.97(0.08)
Boreal	NEE	0.16	127.27(0.45)	59.98(0.81)	3.59(0.17)
Mediterranean	NEE	0.20	199.63(18.51)	96.70(10.24)	-0.71(0.38)

Cambial activity and xylem formation

Cambial activity and wood formation stages were fitted with skewed normal distribution curves with a residual standard error (RSE) ranging between 0.14 and 0.26 (Supplementary Table 3). For cambial activity and cells differentiation stages, the peak was reached later in the boreal biome, and the period of maximum activity was shorter than in temperate and Mediterranean biomes that followed in this specific order (Supplementary Figure 4). Cambial activity in boreal and temperate ecosystems showed a peak at the beginning of summer and end of spring, respectively. In the Mediterranean biome, the peak was substantially earlier during spring. Cell differentiation showed a peak in the number of cells around the summer solstice (i.e., middle of June – start of July), with cell enlargement peaks localized in mid-June and cell wall thickening and lignification peaks localized at the beginning of July (Supplementary Figure 4).

The Mediterranean climate is characterised by dry and hot summers, and optimal growth conditions occur during the cooler and rainy springs and autumns. Being sensitive to drought, radial growth may cease in response to water shortage (Muller et al., 2011), leading to a bimodal growth pattern with a temporary cessation or reduction of radial growth in summer (Camarero et al., 2010). For this reason, we tried to fit a bimodal curve for the Mediterranean biome, but, finally, comparing the fittings, it was decided to use a skewed normal distribution that guaranteed a better fitting. Growth resumption in autumn after summer cease in response to drought is a plastic adaptation to large spatiotemporal variability in the climatic conditions in the Mediterranean region. However, even if the drought period can be temporally localized during a broad time window such as the summer season, the exact period when a moisture shortage comes into play can vary spatially and year by year in the Mediterranean region (Martins et al., 2012; Ruiz-Sinoga et al., 2012). Moreover, it is largely recognised that different species can show different responses to the dry period (Camarero et al., 2010). For all these reasons, in this context, by considering several species and different years, it was unlikely to highlight a bimodal growth pattern in the Mediterranean biome.

Supplementary Table 4 Maximum value (Max), onset, ending and duration of the period of maximum activity (MA), total and period of maximum activity area under the curve (AUC) and their ratio for all the curves describing the seasonal dynamics of wood formation (i.e., cambial activity, cell enlargement, and cell wall thickening and lignification).

Wood formation								
Biome	Process	Max (DOY)	Onset MA (DOY)	Ending MA (DOY)	Duration MA (days)	Total AUC	AUC MA	AUC MA / Total AUC (%)
Boreal	Cambial activity	168.13	150.20	194.61	44.41	62.61	32.53	51.96
Temperate	Cambial activity	152.18	124.75	188.77	64.02	85.53	44.94	52.54
Mediterranean	Cambial activity	107.34	36.41	205.13	168.72	151.93	96.42	63.46
Boreal	Cell enlargement	174.53	159.66	194.47	34.81	51.73	27.16	52.50
Temperate	Cell enlargement	173.35	147.61	199.35	51.74	68.77	37.90	55.11
Mediterranean	Cell enlargement	164.50	125.04	203.85	78.81	95.85	52.95	55.24
Boreal	Cell wall thickening and lignification	203.80	182.63	227.78	45.15	69.25	37.31	53.88
Temperate	Cell wall thickening and lignification	193.47	170.31	232.76	62.45	89.56	48.85	54.54
Mediterranean	Cell wall thickening and lignification	182.95	155.23	239.32	84.09	103.92	56.27	54.15

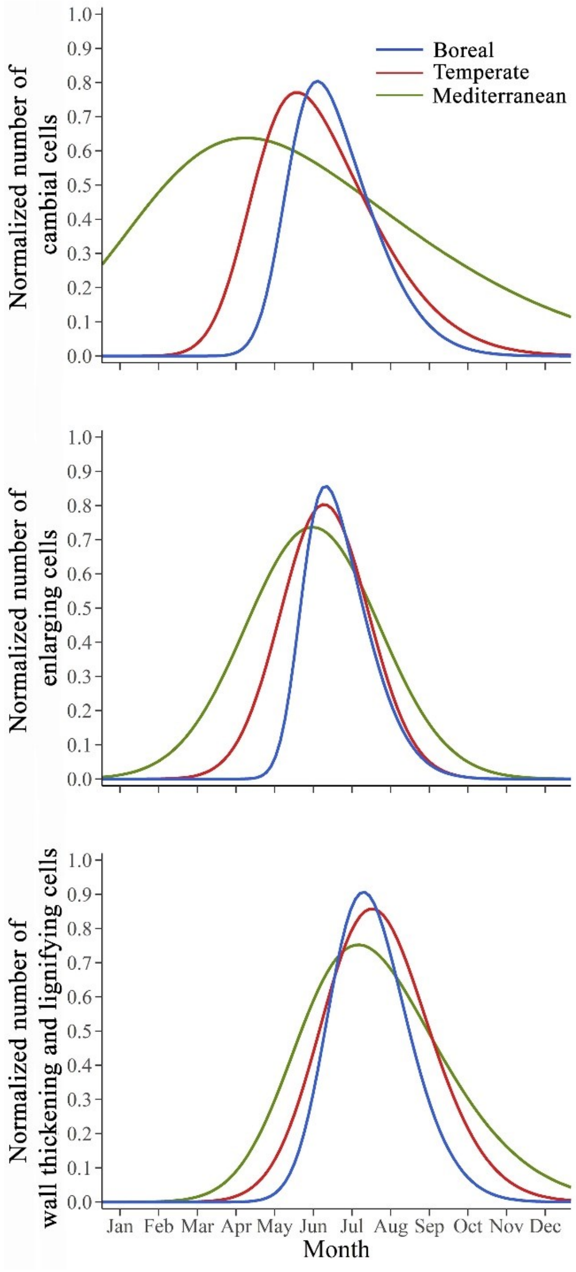

Supplementary Figure 4 Seasonal pattern of cambial activity and cell differentiation stages (i.e., enlargement and secondary wall thickening and lignification), normalized according to the biome and study site in conifers as a function of the biome (i.e., Boreal, Temperate and Mediterranean).

NSC

Seasonal oscillations of starch and soluble sugars for all organs in all biomes showed a residual standard error (RSE) ranging between 0.10 and 0.27 (Supplementary Table 3). The seasonal patterns of starch and soluble sugars were characterised by opposite temporal dynamics, particularly evident in needles with a very sharp starch peak in late spring-early summer strictly followed by the seasonal minimum for soluble sugars (Supplementary Figure 5). The amplitude of the oscillations among biomes was most prominent for starch and particularly noticeable in needles. Starch levels peaked belowground first (~early spring), then in stems (mid-spring) and finally in needles (late spring-early summer) but not regarding the boreal biome where the peak of starch in roots was later than the peak in needles and stem. Soluble sugars in roots and stems were less variable, with a hint of a seasonal minimum around late spring-early summer (Supplementary Figure 5). However, soluble sugar concentration in roots showed a peak in autumn in the Mediterranean biome. Boreal and temperate ecosystems showed contrasting temporal dynamics for starch and soluble sugar. Starch peaked in needles, stems and roots around late spring-early summer, mid-spring, and midsummer, respectively. In contrast, soluble sugars were lowest in all organs from late spring to midsummer. Temperate biomes were characterised by maximum starch concentrations towards late spring-early summer, particularly in needles, strictly followed by minimum levels of soluble sugars in all organs. In the Mediterranean, the magnitude of such oscillation was generally lower than those observed for boreal and temperate ecosystems (Supplementary Figure 5).

Supplementary Table 5 Maximum value (Max), onset, ending and duration of the period of maximum activity (MA), total and period of maximum activity area under the curve (AUC) and their ratio for all the curves describing the seasonal dynamics of NSC (i.e., starch and soluble sugars in needles, stem and roots).

Non structural carbohydrates								
Biome	Process	Max (month)	Onset MA (month)	Ending MA (month)	Duration MA (months)	Total AUC	AUC MA	AUC MA / Total AUC (%)
Boreal	Needles starch	5.78	4.77	6.95	2.18	2.05	1.10	53.66
Temperate	Needles starch	5.45	3.98	7.01	3.03	2.65	1.46	55.09
Mediterranean	Needles starch	4.79	3.03	7.55	4.52	1.03	0.66	64.08
Boreal	Needles soluble sugars	6.89	3.45	10.07	6.62	6.03	2.99	49.59
Temperate	Needles soluble sugars	6.99	3.95	11.26	7.31	5.53	3.36	60.76
Mediterranean	Needles soluble sugars	5.52	2.40	8.63	6.23	4.57	2.46	53.83
Boreal	Stem starch	5.89	3.75	8.02	4.27	2.45	1.32	53.88
Temperate	Stem starch	5.13	2.95	7.58	4.63	2.90	1.79	61.72
Mediterranean	Stem starch	4.61	1.97	7.26	5.29	2.39	1.47	61.51
Boreal	Stem soluble sugars	6.91	3.99	9.75	5.76	3.01	1.29	42.86
Temperate	Stem soluble sugars	6.46	3.34	9.28	5.94	4.19	2.09	49.88
Mediterranean	Stem soluble sugars	6.25	3.08	9.42	6.34	3.74	1.83	48.93
Boreal	Roots starch	6.97	4.93	9.01	4.08	2.07	1.10	53.14
Temperate	Roots starch	4.58	2.38	7.12	4.74	4.29	2.35	54.78
Mediterranean	Roots starch	3.94	1.99	5.51	3.52	4.76	3.14	65.97
Boreal	Roots soluble sugars	7.03	3.42	10.64	7.22	4.39	2.31	52.62
Temperate	Roots soluble sugars	6.39	3.38	9.39	6.01	4.39	2.08	47.38
Mediterranean	Roots soluble sugars	9.63	7.07	11.24	4.17	6.74	3.85	57.12

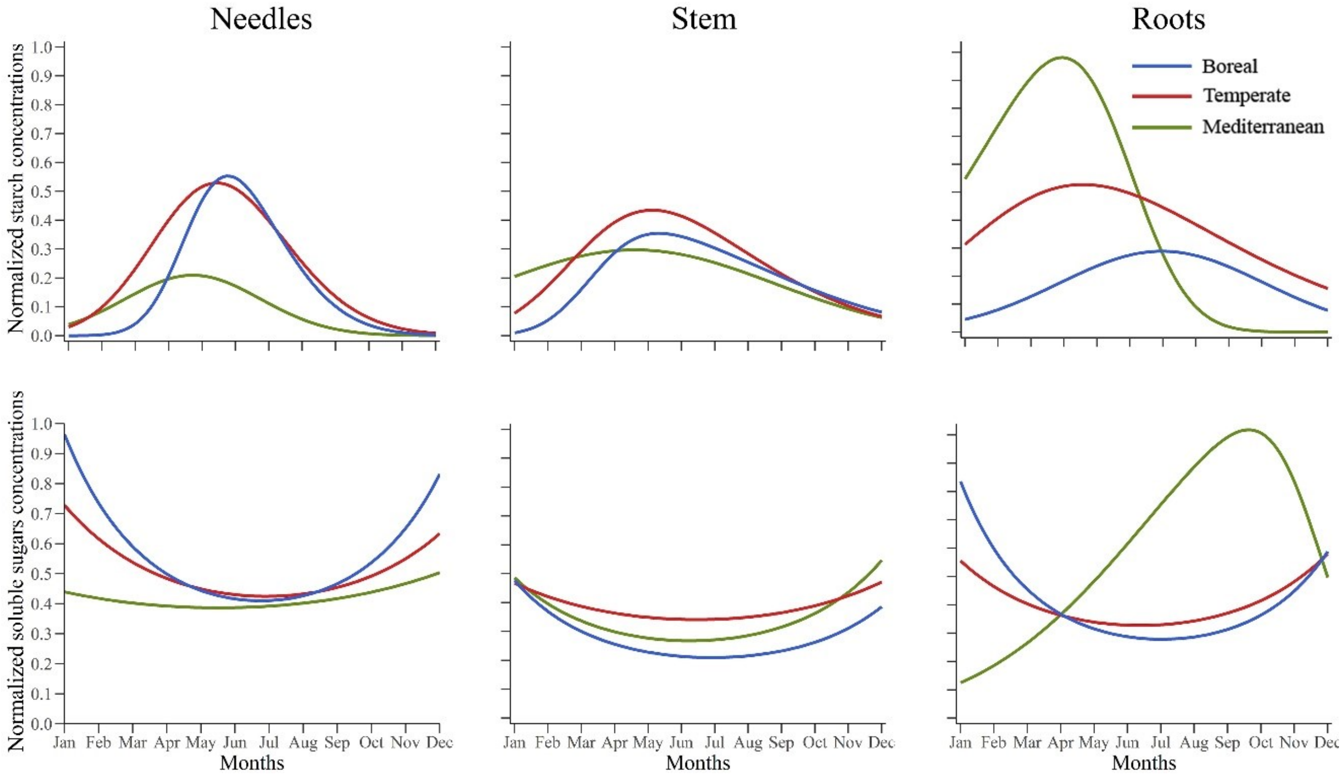

Supplementary Figure 5 Seasonal dynamics of NSC concentration (i.e., starch and soluble sugars, normalized according to biome, organ, study and quantification method) in conifers as a function of organ (i.e., needles, stem, roots) and biome (i.e., Boreal, Temperate and Mediterranean).

Carbon fluxes

All carbon flux dynamics (i.e., NEE, GPP, RECO) were fitted with skewed normal distribution curves with a residual standard error (RSE) ranging between 0.09 and 0.20 (Supplementary Table 3). For each flux, in the boreal biome the peak was reached later, and the period of maximum activity was shorter, generally starting later and ending earlier than temperate and Mediterranean biomes that followed in this specific order (Supplementary Figure 6). The NEE peak was earlier than RECO and GPP peaks for each biome and reached a maximum at the end of spring between May and June. RECO and GPP peaked later during summer, with the peak of RECO following the peak of GPP by about two weeks in each biome (Supplementary Figure 6).

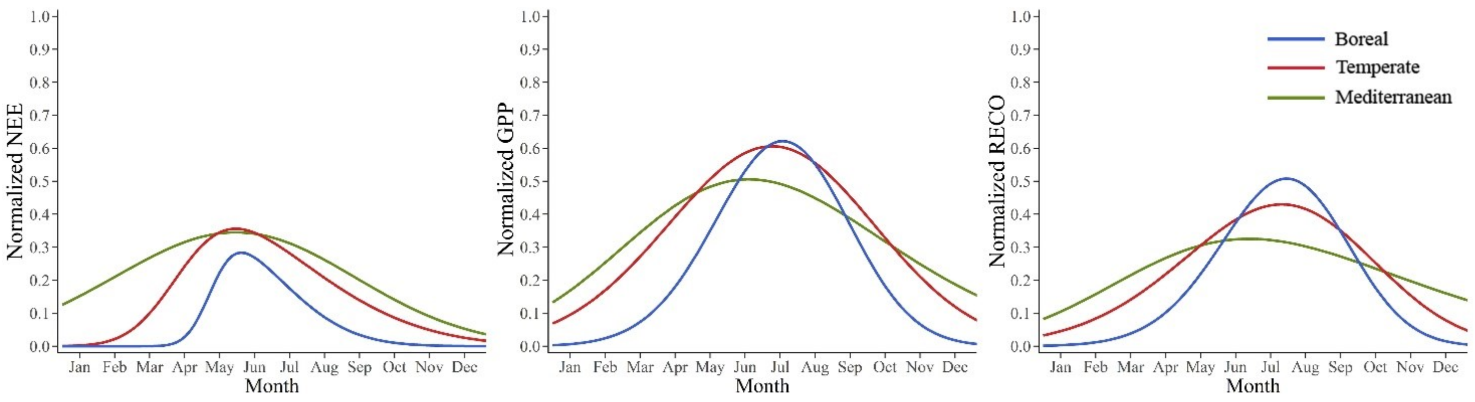

Supplementary Figure 6 Seasonal pattern of Net Ecosystem Exchange (NEE), Ecosystem Respiration (RECO), and Gross Primary Production (GPP), normalized according to the biome, flux type (i.e., NEE, RECO and GPP) and study site in conifers as a function of the biome (i.e., Boreal, Temperate and Mediterranean).

Supplementary Table 6 Maximum value (Max), onset, ending and duration of the period of maximum activity (MA), total and period of maximum activity area under the curve (AUC) and their ratio for all the curves describing the seasonal dynamics of carbon fluxes (i.e., NEE, GPP and RECO).

C fluxes								
**Biome**	**Process**	**Max (DOY)**	**Onset MA (DOY)**	**Ending MA (DOY)**	**Duration MA (days)**	**Total AUC**	**AUC MA**	**AUC MA / Total AUC (%)**
Boreal	GPP	197.28	153.85	239.76	85.91	89.23	48.79	54.68
Temperate	GPP	188.16	125.51	250.03	124.52	128.43	64.42	50.16
Mediterranean	GPP	168.22	103.51	238.56	135.05	129.03	69.68	54.00
Boreal	RECO	208.72	166.78	248.83	82.05	79.87	45.94	57.52
Temperate	RECO	204.51	145.47	260.22	114.75	85.81	43.07	50.19
Mediterranean	RECO	176.51	107.47	255.67	148.20	86.46	45.43	52.54
Boreal	NEE	153.59	133.64	183.42	49.78	24.91	12.85	51.59
Temperate	NEE	148.76	110.63	200.49	89.86	55.38	29.14	52.62
Mediterranean	NEE	147.58	94.47	219.73	125.26	80.57	40.58	50.37

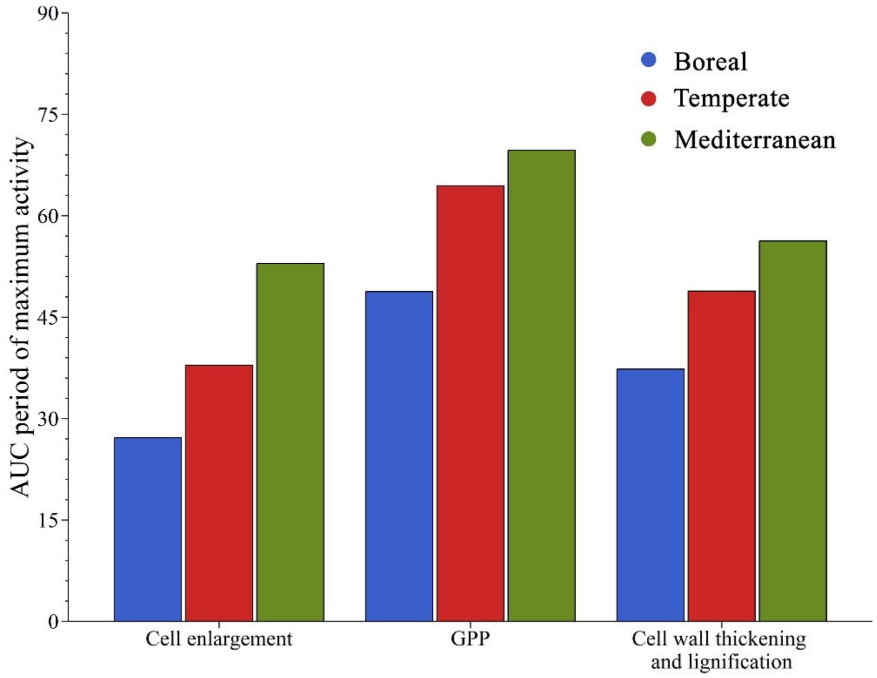

Supplementary Figure 7 Area under the curve (AUC) of the period of maximum activity (MA) of the processes of Gross Primary Production (GPP) and phenological phases of cell enlargement and cell wall thickening and lignification during wood formation in boreal, temperate and Mediterranean biomes.

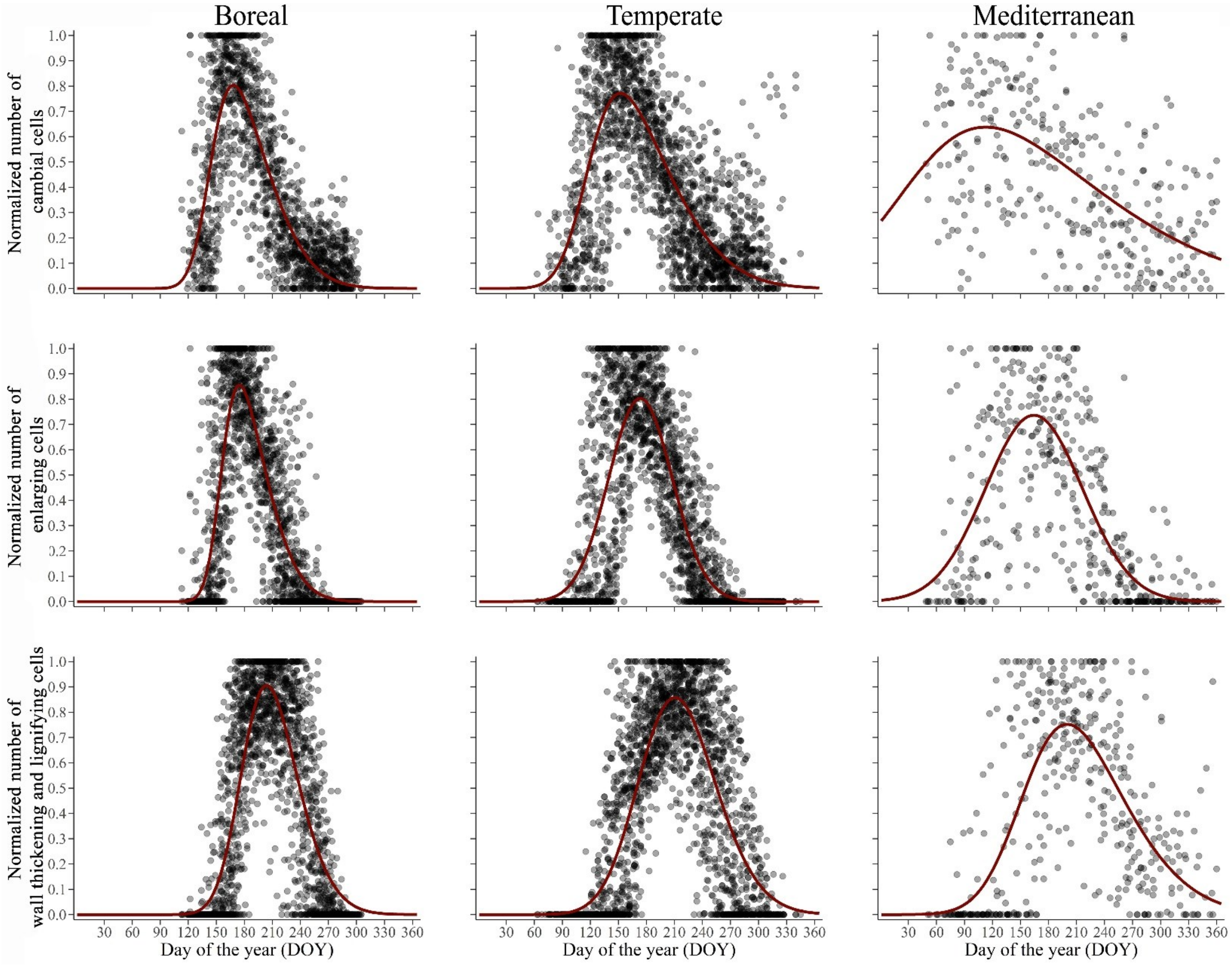

Supplementary Figure 8 Fitting details for cambial activity and cell differentiation phenological phase in boreal, temperate and Mediterranean biomes.

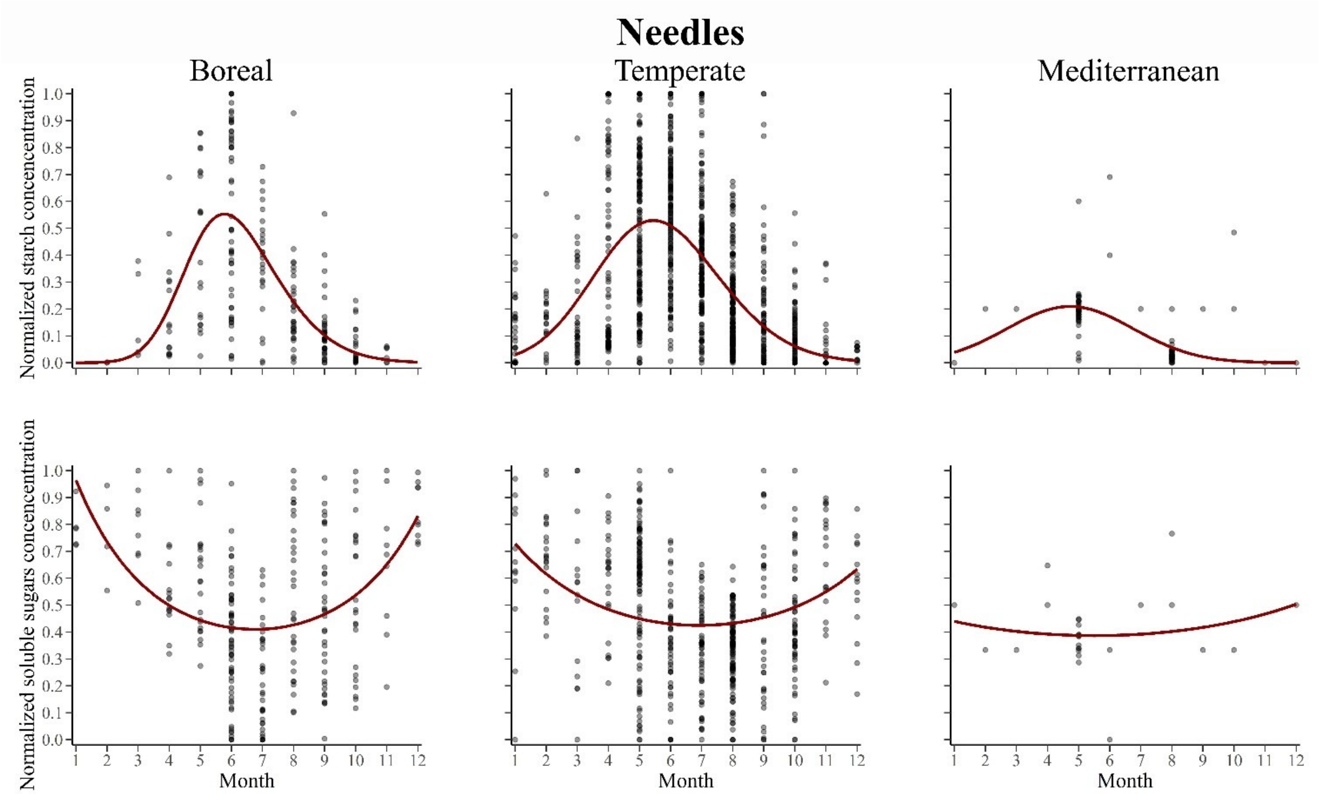

Supplementary Figure 9 Fitting details for starch and soluble sugars in needles in boreal, temperate and Mediterranean biomes.

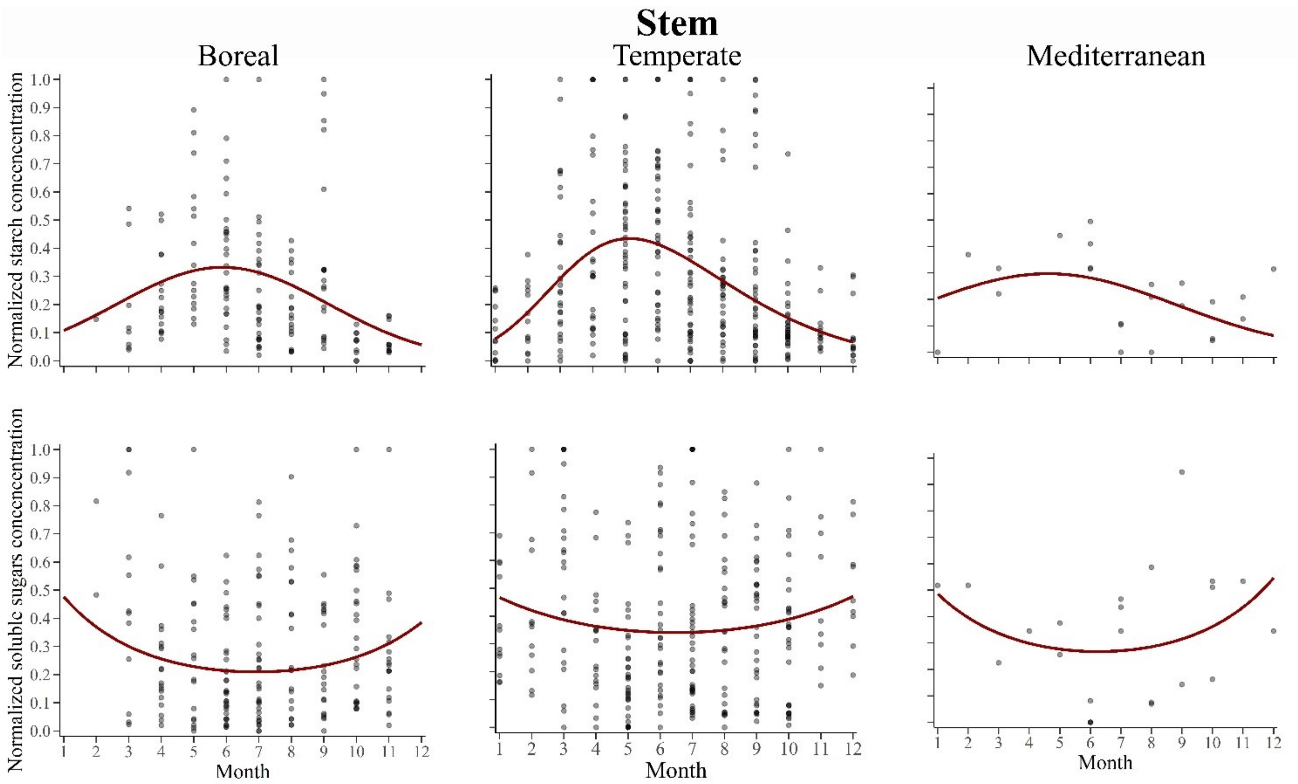

Supplementary Figure 10 Fitting details for starch and soluble sugars in stem in boreal, temperate and Mediterranean biomes.

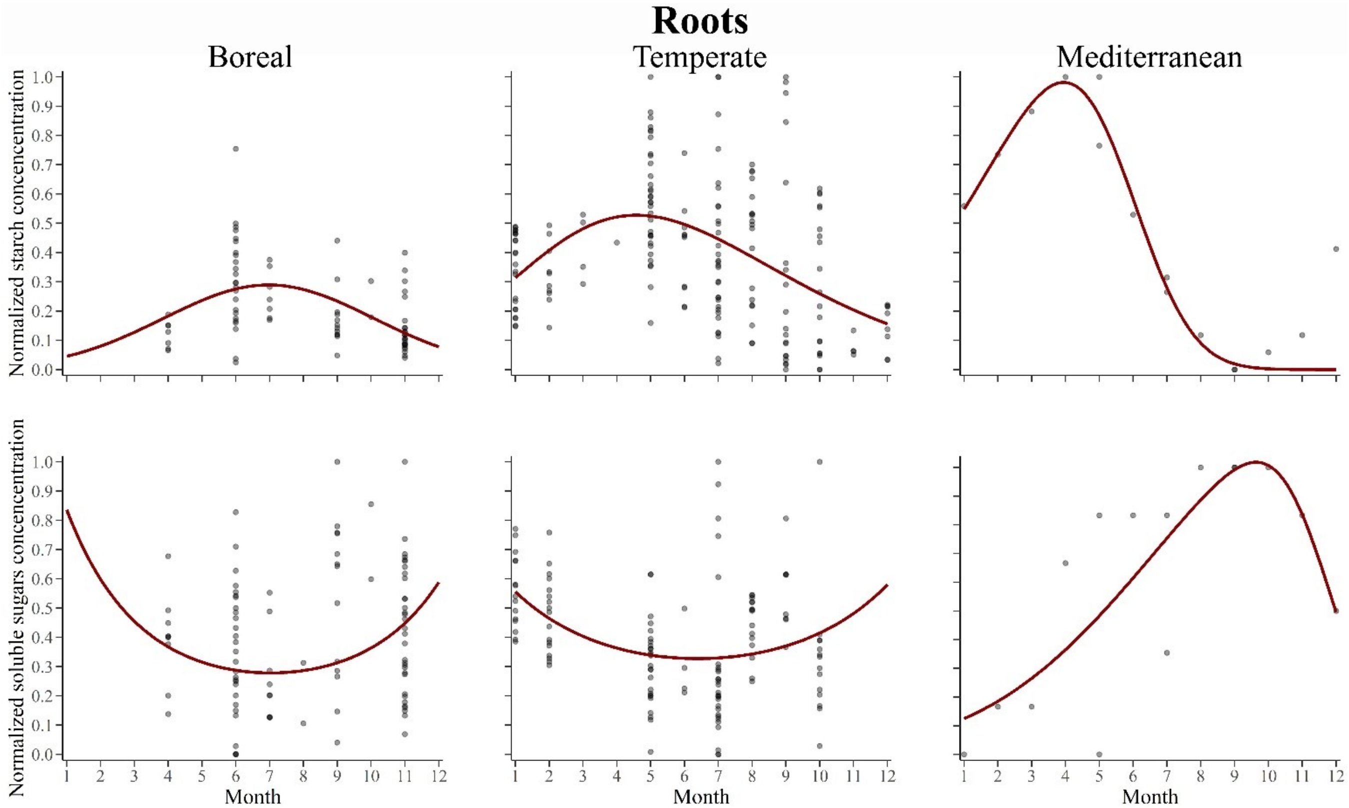

Supplementary Figure 11 Fitting details for starch and soluble sugars in roots in boreal, temperate and Mediterranean biomes.

Seasonal carbon fluxes

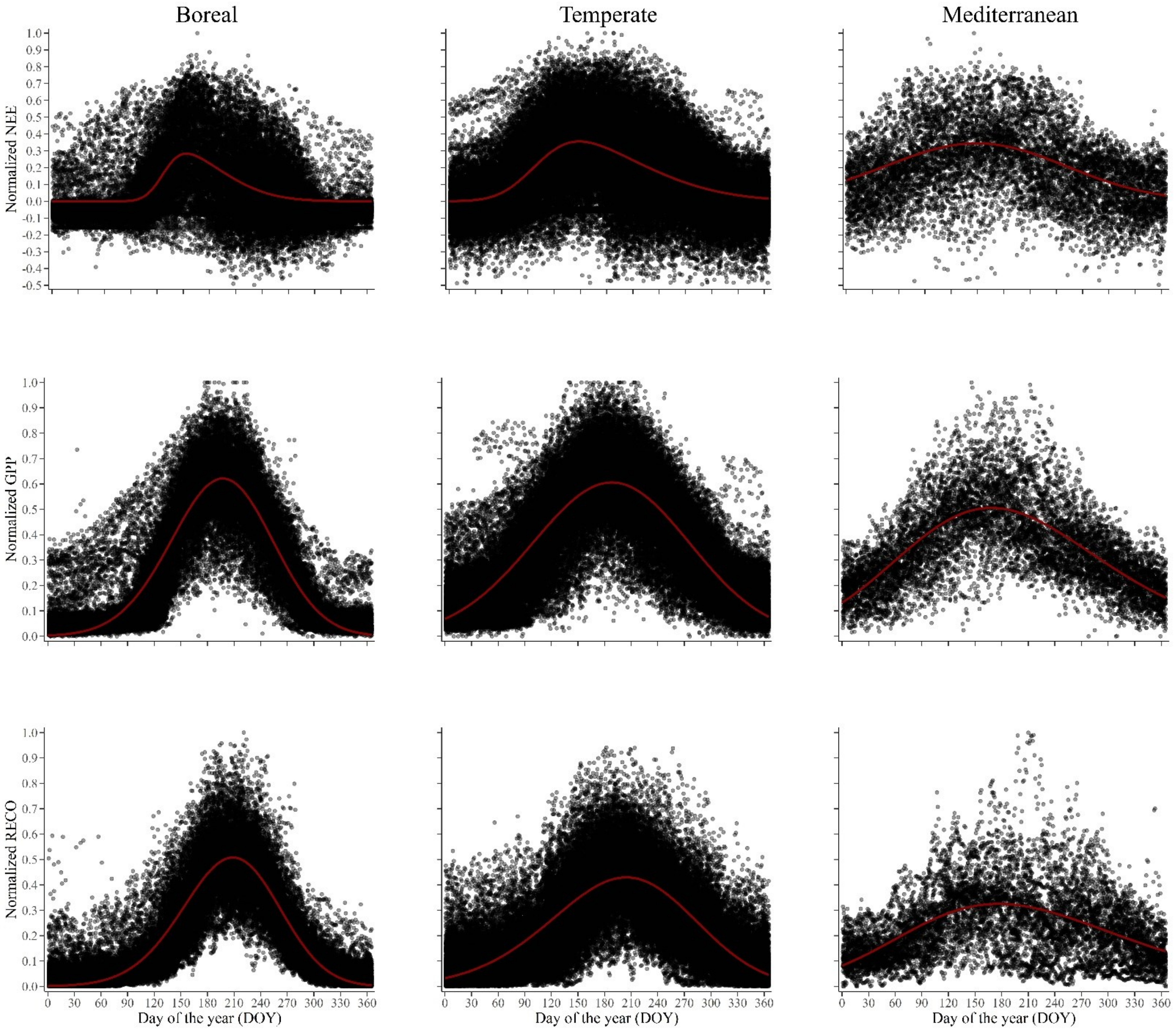

Supplementary Figure 12 Fitting details for Net Ecosystem Exchange (NEE), Ecosystem Respiration (RECO) and Gross Primary Production (GPP) in boreal, temperate and Mediterranean biomes.

Supplementary Note 1: SMA regressions following bioclimatic classification.

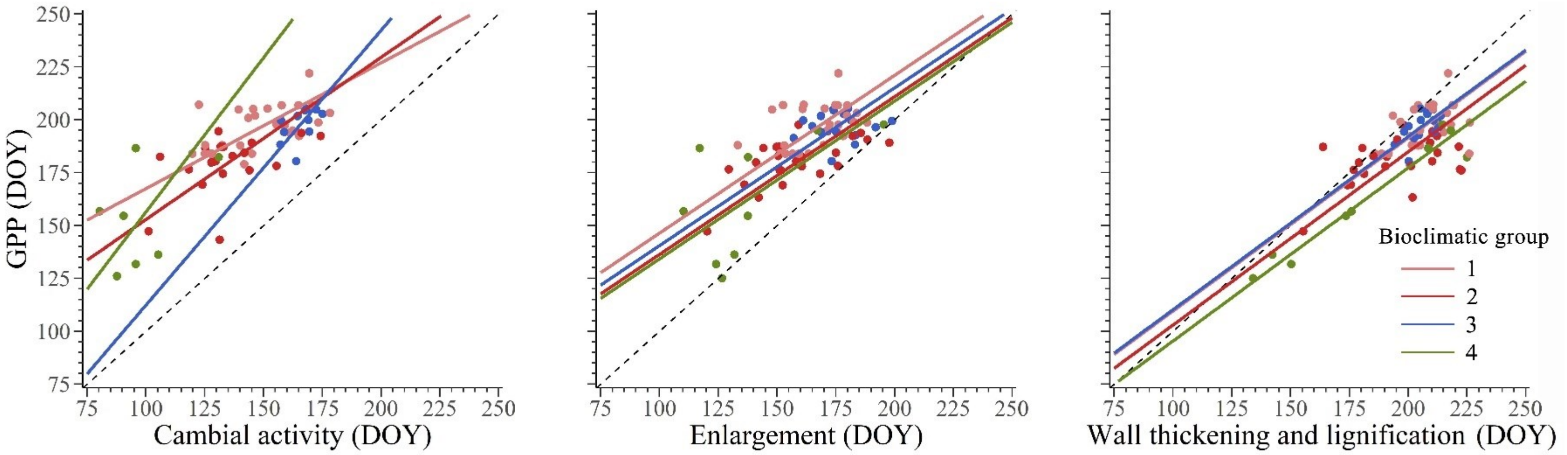

Supplementary Figure 13 Synchronisms among the timing of peak of Gross Primary Production (GPP) and phenological phases of wood formation (i.e., cambial activity, cell enlargement, and cell wall thickening and lignification) in 87 sites across boreal (1, blue lines and symbols), temperate (2 and 3, green lines and symbols) and Mediterranean (4, red lines and symbols) biomes. The dashed line represents a bisecting line (1:1).

Supplementary Table 7 Results of Standardized Major Axis (SMA) analyses of the bivariate relationships among timing of the peak of GPP and timing of cambial activity, cell enlargement and cell wall thickening and lignification in 81 sites according to their bioclimatic classification. * Indicates non-significant ($p > 0.05$) regressions.

Phenological stage	Bioclimatic group	Y-intercept	Slope	95% CI slope	R^2
Cambial activity	1	107.95	0.59	0.43 - 0.82	0.32
	2	76.12	0.77	0.50 - 1.18	0.24
	3	-17.95	1.30	0.70 - 2.40	0.12*
	4	10.73	1.46	0.59 - 3.61	0.11*
Cell enlargement	1	71.74	0.75	0.60 - 0.91	0.2
	2	61.67	0.75	0.60 - 0.91	0.38
	3	65.83	0.75	0.60 - 0.91	0.37
	4	59.57	0.75	0.60 - 0.91	0.36
Cell wall thickening and lignification	1	27.65	0.82	0.71 - 0.96	0.27
	2	21.07	0.82	0.71 - 0.96	0.22
	3	28.31	0.82	0.71 - 0.96	0.35
	4	13.51	0.82	0.71 - 0.96	0.94

Supplementary Note 3: Random forest regression models

Supplementary Table 8 Random forest regression models applied to FluxNET (i.e., NEE, GPP, RECO), FluxSat (i.e., GPP), cambial activity and xylem cell differentiation (i.e., cell enlargement and cell wall thickening and lignification timings of peaks. For each model, the table show the percentage of variance explained, the root mean squared error (RMSE) and the R^2 for both the traing and test set.

	Process	Variance explained (%)	RMSE	R^2 (training set)	R^2 (test set)
**C Flux**	NEE (FluxNET)	44.09	37.9	0.86	0.68
	GPP (FluxNET)	27.86	18.71	0.81	0.51
	RECO (FluxNET)	23.56	22.92	0.72	0.49
	GPP (FluxSat)	54.06	21.01	0.89	0.66
**Wood formation**	Cambial activity	43.09	18.68	0.83	0.7
	Cell enlargement	38.09	27.26	0.76	0.83
	Cell wall thickening and lignification	39.96	26.39	0.76	0.73

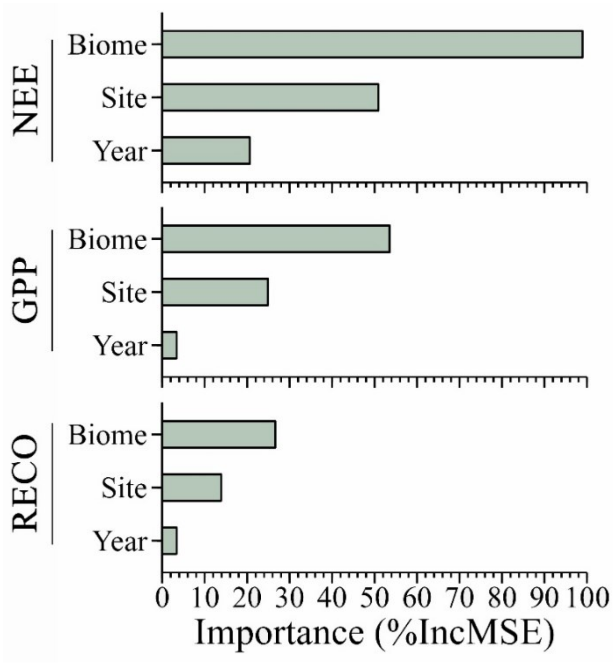

Supplementary Figure 14 Relative importance in terms of Mean Decrease Accuracy (%IncMSE) of predictors in the random forest regression models for the timing of peak of FluxNET Net Ecosystem Exchange (NEE), Gross Primary Production (GPP), and Ecosystem Respiration (RECO).

Supplementary References

Camarero, J. J., Olano, J. M., & Parras, A. (2010). Plastic bimodal xylogenesis in conifers from continental Mediterranean climates. *New Phytologist*, *185*(2), 471-480. https://doi.org/10.1111/j.1469-8137.2009.03073.x

Joiner, J., & Yoshida, Y. (2021). Global MODIS and FLUXNET-derived Daily Gross Primary Production, V2. *ORNL DAAC*. https://doi.org/10.3334/ORNLDAAC/1835

Martínez-Vilalta, J., Sala, A., Asensio, D., Galiano, L., Hoch, G., Palacio, S., Piper, F. I., & Lloret, F. (2016). Dynamics of non-structural carbohydrates in terrestrial plants: a global synthesis. *Ecological Monographs*, *86*(4), 495-516. https://doi.org/10.1002/ecm.1231

Martins, D. S., Raziei, T., Paulo, A. A., & Pereira, L. S. (2012). Spatial and temporal variability of precipitation and drought in Portugal. *Natural Hazards and Earth System Science*, *12*(5), 1493-1501. https://doi.org/10.5194/nhess-12-1493-2012

Muller, B., Pantin, F., Génard, M., Turc, O., Freixes, S., Piques, M., & Gibon, Y. (2011). Water deficits uncouple growth from photosynthesis, increase C content, and modify the relationships between C and growth in sink organs. *Journal of Experimental Botany*, *62*(6), 1715-1729. https://doi.org/10.1093/jxb/erq438

Pastorello, G., Trotta, C., Canfora, E., Chu, H., Christianson, D., Cheah, Y. W., Poindexter, C., Chen, J., Elbashandy, A., Humphrey, M., Isaac, P., Polidori, D., Ribeca, A., van Ingen, C., Zhang, L., Amiro, B., Ammann, C., Arain, M. A., Ardö, J., . . . Papale, D. (2020). The FLUXNET2015 dataset and the ONEFlux processing pipeline for eddy covariance data. *Scientific Data*, *7*(1), 225-225. https://doi.org/10.1038/s41597-020-0534-3

Quentin, A. G., Pinkard, E. A., Ryan, M. G., Tissue, D. T., Baggett, L. S., Adams, H. D., Maillard, P., Marchand, J., Landhäusser, S. M., Lacointe, A., Gibon, Y., Anderegg, W. R. L., Asao, S., Atkin, O. K., Bonhomme, M., Claye, C., Chow, P. S., Clément-Vidal, A., Davies, N. W., . . . Woodruff, D. R. (2015). Non-structural carbohydrates in woody plants compared among laboratories. *Tree*

Physiology, *35*(11), 1146-1165. https://doi.org/10.1093/treephys/tpv073

Rossi, S., Anfodillo, T., & Menardi, R. (2006). Trephor: A new tool for sampling microcores from tree stems. *IAWA Journal*, *27*(1), 89-97. https://doi.org/10.1163/22941932-90000139

Rossi, S., Deslauriers, A., & Anfodillo, T. (2006). Assessment of cambial activity and xylogenesis by microsampling tree species: An example at the Alpine timberline. *IAWA Journal*, *27*(4), 383-394. https://doi.org/10.1163/22941932-90000161

Ruiz-Sinoga, J. D., Garcia-Marin, R., Gabarron-Galeote, M. A., & Martinez-Murillo, J. F. (2012). Analysis of dry periods along a pluviometric gradient in Mediterranean southern Spain. *International Journal of Climatology*, *32*(10), 1558-1571. https://doi.org/10.1002/joc.2376
