# Supplementary material for: Partial asynchrony of coniferous forest carbon sources and sinks at the intra-annual time scale
Source: Nat Commun. 2024 Aug 5;15:6169. doi: 10.1038/s41467-024-49494-5 (PMC11300610; doi:10.1038/s41467-024-49494-5)
Supplement: Supplementary file 3 — Description of Additional Supplementary Files [file 41467_2024_49494_MOESM3_ESM.pdf]

Description of Additional Supplementary Files

File Name: Supplementary Data 1
Description: Geographical coordinates and study period for wood formation sites; Geographical coordinates and references for non-structural carbohydrates study sites; Geographical coordinates and hyperlinks for FluxNet sites.

File Name: Supplementary Data 2
Description: Delta (i.e., subtraction) between the timing of 10th, 25th, 50th, 75th, 90th percentile and peak (i.e., 100th percentile) among C fluxes, i.e., NEE, GPP, RECO and phenological phases of wood formation, i.e., cambial activity, cell enlargement and cell wall thickening and lignification in boreal, temperate and Mediterranean biomes.
