# Supplementary material for: Partial asynchrony of coniferous forest carbon sources and sinks at the intra-annual time scale
Source: Nat Commun. 2024 Aug 5;15:6169. doi: 10.1038/s41467-024-49494-5 (PMC11300610; doi:10.1038/s41467-024-49494-5)
Supplement: Supplementary file 6 — Reporting Summary [file 41467_2024_49494_MOESM6_ESM.pdf]

nature portfolio

Corresponding author(s): Roberto Silvestro

Last updated by author(s): Apr 30, 2024

Reporting Summary

Nature Portfolio wishes to improve the reproducibility of the work that we publish. This form provides structure for consistency and transparency in reporting. For further information on Nature Portfolio policies, see our Editorial Policies and the Editorial Policy Checklist.

Statistics

For all statistical analyses, confirm that the following items are present in the figure legend, table legend, main text, or Methods section.

n/a	Confirmed	
☐	☒	The exact sample size (*n*) for each experimental group/condition, given as a discrete number and unit of measurement
☐	☒	A statement on whether measurements were taken from distinct samples or whether the same sample was measured repeatedly
☐	☒	The statistical test(s) used AND whether they are one- or two-sided<br>*Only common tests should be described solely by name; describe more complex techniques in the Methods section.*
☒	☐	A description of all covariates tested
☐	☒	A description of any assumptions or corrections, such as tests of normality and adjustment for multiple comparisons
☐	☒	A full description of the statistical parameters including central tendency (e.g. means) or other basic estimates (e.g. regression coefficient) AND variation (e.g. standard deviation) or associated estimates of uncertainty (e.g. confidence intervals)
☐	☒	For null hypothesis testing, the test statistic (e.g. *F*, *t*, *r*) with confidence intervals, effect sizes, degrees of freedom and *P* value noted<br>*Give P values as exact values whenever suitable.*
☒	☐	For Bayesian analysis, information on the choice of priors and Markov chain Monte Carlo settings
☒	☐	For hierarchical and complex designs, identification of the appropriate level for tests and full reporting of outcomes
☒	☐	Estimates of effect sizes (e.g. Cohen's *d*, Pearson's *r*), indicating how they were calculated

Our web collection on statistics for biologists contains articles on many of the points above.

Software and code

Policy information about availability of computer code

Data collection

For wood formation have been used data from 81 study sites. The sample size ranged from 1 to 55 trees among all sites throughout the entire growing seasons of 1998 to 2018. Stem microcores were collected weekly, or occasionally biweekly, at breast height (i.e., 1.3 m) using surgical bone-sampling needles or a Trephor tool. The samples included mature and developing xylem of the current year, the cambial zone and adjacent phloem, and at least one previous complete tree ring.

We assembled the NSC dataset by sub-setting the conifers section of the dataset available at Dryad: http://dx.doi.org/10.5061/dryad.j6r5k, finally resulting in 57 sites distributed in boreal, temperate and Mediterranean biomes. The studies included seasonal NSC data on wild species measured under natural field conditions. When studies involved experimental manipulations, we only considered results from unmanipulated controls. In addition, to ensure good temporal coverage and reduce unwanted variability due to some specific characteristics of the samples, we selected only work that fulfilled the following criteria: (1) study duration was at least four months, (2) the same individuals or populations were measured at least three times spanning the length of the study, (3) plants were mature, (4) measurements were taken on needles, main stem, fine or coarse roots (5) values reported were starch/fructans, or soluble sugars.

We used tier-one level data from the FLUXNET2015 dataset (https://fluxnet.org/data/fluxnet2015-dataset/) and extracted data at daily temporal aggregation from ENF (Evergreen Needleleaf Forests) sites. These sites consist of forest lands dominated by woody vegetation with a cover of >60% and height exceeding 2 meters. In addition, to reduce unwanted variability due to some specific characteristics of the site, we selected only data in which stands (1) belonged to boreal, temperate, or Mediterranean biomes, (2) were at least 15 years old, and (3) were not recently disturbed (e.g., burn sites). The dataset finally consisted of 39 sites. CO2 fluxes extracted for each site were Net Ecosystem Exchange (NEE), Ecosystem Respiration (RECO), and Gross Primary Production (GPP).

Daily GPP data were extracted for each site and each year where the wood formation was monitored. Differently from the dataset obtained

with the data from FLUXNET2015, in this case, while the samples were collected on coniferous species, the study area could consist of a mixed forest. These GPP products were extracted by FluxSat v2.0 (https://daac.ornl.gov/cgi-bin/dsviewer.pl?ds_id=1835), where FluxSat refers to data derived using FLUXNET eddy covariance tower site data and the coincident satellite data. R has been used to automatically filter Fluxsat dataset.

Data analysis

All the statistics have been performed in R version 4.2.2 using:

- "randomForest" package for random forest models.
- "smatr" package for standardized major axis regressions.
- "nls.multistat" package for non-linear fittings.
- "factomineR" and "factoextra" packages for bioclimatic analysis.

For manuscripts utilizing custom algorithms or software that are central to the research but not yet described in published literature, software must be made available to editors and reviewers. We strongly encourage code deposition in a community repository (e.g. GitHub). See the Nature Portfolio guidelines for submitting code & software for further information.

Data

Policy information about availability of data

All manuscripts must include a data availability statement. This statement should provide the following information, where applicable:

- Accession codes, unique identifiers, or web links for publicly available datasets
- A description of any restrictions on data availability
- For clinical datasets or third party data, please ensure that the statement adheres to our policy

Data generated in this study have been deposited in Borealis: http://doi.org/10.5683/SP3/JRDOU1. Wood formation raw data are available under restricted access and can be accessed directly contacting the corresponding author of this study or using the procedure in Borealis. Data on non-structural carbohydrates are available at Dryad: http://dx.doi.org/10.5061/dryad.j6r5k. Access to FluxNet data is provided through the FluxNet portal: https://fluxnet.org/data/fluxnet2015-dataset/. FluxSat data can be accessed via the FluxSat portal: https://daac.ornl.gov/cgi-bin/dsviewer.pl?ds_id=1835. Details of all sites used for the non-structural carbohydrates data, FluxNet data, and geographical coordinates for downloading FluxSat data are listed in the supplementary methods section.

Research involving human participants, their data, or biological material

Policy information about studies with human participants or human data. See also policy information about sex, gender (identity/presentation), and sexual orientation and race, ethnicity and racism.

Reporting on sex and gender	n/a
Reporting on race, ethnicity, or other socially relevant groupings	n/a
Population characteristics	n/a
Recruitment	n/a
Ethics oversight	n/a

Note that full information on the approval of the study protocol must also be provided in the manuscript.

Field-specific reporting

Please select the one below that is the best fit for your research. If you are not sure, read the appropriate sections before making your selection.

☐ Life sciences ☐ Behavioural & social sciences ☒ Ecological, evolutionary & environmental sciences

For a reference copy of the document with all sections, see nature.com/documents/nr-reporting-summary-flat.pdf

Ecological, evolutionary & environmental sciences study design

All studies must disclose on these points even when the disclosure is negative.

Study description

The present study is a data synthesis that assesses the temporal relationship between carbon sources and sinks in forest ecosystems. We analyzed data from 177 sites in the northern hemisphere, which included measurements related to the temporal dynamics of wood formation, non-structural carbohydrates (NSC), and carbon fluxes. Therefore, this study is based entirely on previously published data.

Research sample

The study have been performed on coniferous species belonging to boreal, temperate, and mediterranean biomes of the northern hemiphere.

For wood formation have been used data from 81 study sites. The sample size ranged from 1 to 55 trees among all sites throughout the entire growing seasons of 1998 to 2018. Stem microcores were collected weekly, or occasionally biweekly, at breast height (i.e., 1.3 m) using surgical bone-sampling needles or a Trephor tool. The samples included mature and developing xylem of the current year, the cambial zone and adjacent phloem, and at least one previous complete tree ring.

We assembled the NSC dataset by sub-setting the conifers section of the dataset available at Dryad: http://dx.doi.org/10.5061/dryad.j6r5k, finally resulting in 57 sites distributed in boreal, temperate and Mediterranean biomes. The studies included seasonal NSC data on wild species measured under natural field conditions. When studies involved experimental manipulations, we only considered results from unmanipulated controls. In addition, to ensure good temporal coverage and reduce unwanted variability due to some specific characteristics of the samples, we selected only work that fulfilled the following criteria: (1) study duration was at least four months, (2) the same individuals or populations were measured at least three times spanning the length of the study, (3) plants were mature, (4) measurements were taken on needles, main stem, fine or coarse roots (5) values reported were starch/fructans, or soluble sugars.

We used tier-one level data from the FLUXNET2015 dataset (https://fluxnet.org/data/fluxnet2015-dataset/) and extracted data at daily temporal aggregation from ENF (Evergreen Needleleaf Forests) sites. These sites consist of forest lands dominated by woody vegetation with a cover of >60% and height exceeding 2 meters. In addition, to reduce unwanted variability due to some specific characteristics of the site, we selected only data in which stands (1) belonged to boreal, temperate, or Mediterranean biomes, (2) were at least 15 years old, and (3) were not recently disturbed (e.g., burn sites). The dataset finally consisted of 39 sites. CO2 fluxes extracted for each site were Net Ecosystem Exchange (NEE), Ecosystem Respiration (RECO), and Gross Primary Production (GPP).

Daily GPP data were extracted for each site and each year where the wood formation was monitored. Differently from the dataset obtained with the data from FLUXNET2015, in this case, while the samples were collected on coniferous species, the study area could consist of a mixed forest. These GPP products were extracted by FluxSat v2.0 (https://daac.ornl.gov/cgi-bin/dsviewer.pl?ds_id=1835), where FluxSat refers to data derived using FLUXNET eddy covariance tower site data and the coincident satellite data.

Sampling strategy	This data synthesis is based on already existing and published data. According to the criteria described in the 'research sample' section, we used all the data we could possibly access.
Data collection	Roberto Silvestro compiled the wood formation dataset using data provided by all the co-authors listed in the author list. The NSC data, along with data from FluxNet and FluxSat, were downloaded in accordance with the criteria outlined in the 'research sample' section, using the links provided in that section.
Timing and spatial scale	This data synthesis is based on already existing and published data. Overall, the sampling period of the data range from 1998 to 2018.
Data exclusions	Data from subtropical sites, although available, were excluded from the analysis because the number of study sites was not considered sufficient to represent the entire biome adequately.
Reproducibility	This data synthesis is based on already existing and published data. Running the same statistical analysis as performed in this study ensures results consistent with those presented.
Randomization	This data synthesis is based on already existing and published data.
Blinding	This data synthesis is based on already existing and published data.

Did the study involve field work? ☐ Yes ☒ No

Reporting for specific materials, systems and methods

We require information from authors about some types of materials, experimental systems and methods used in many studies. Here, indicate whether each material, system or method listed is relevant to your study. If you are not sure if a list item applies to your research, read the appropriate section before selecting a response.

Materials & experimental systems

n/a	Involved in the study	
☒	☐	Antibodies
☒	☐	Eukaryotic cell lines
☒	☐	Palaeontology and archaeology
☒	☐	Animals and other organisms
☒	☐	Clinical data
☒	☐	Dual use research of concern
☐	☒	Plants

Methods

n/a	Involved in the study	
☒	☐	ChIP-seq
☒	☐	Flow cytometry
☒	☐	MRI-based neuroimaging

Plants

Seed stocks	n/a
Novel plant genotypes	n/a
Authentication	n/a
